# Circular RNA circ-ERBB2 promotes HER2-positive breast cancer progression and metastasis via sponging miR-136-5p and miR-198

**DOI:** 10.1186/s12967-021-03114-8

**Published:** 2021-11-03

**Authors:** Jin-xiu Zhong, Yun-yuan Kong, Rong-guang Luo, Guo-jin Xia, Wen-xing He, Xue-zhong Chen, Wei-wei Tan, Qing-jie Chen, Yu-yin Huang, Yan-xing Guan

**Affiliations:** 1 Department of Breast Cancer Center/Nuclear Medicine, The Affiliated Cancer Hospital of Nanchang University, Nanchang, 330029 China; 2 Department of Nuclear Medicine/Radiology, The First Affiliated Hospital of Nanchang University, No. 17 Yong Wai Street, Nanchang, 330006 Jiangxi China

**Keywords:** Circ-ERBB2, miR-136-5p, miR-198, TFAP2C, HER2-positive breast cancer

## Abstract

**Background:**

Circular RNAs (circRNAs) are pivotal regulators of various human cancers and circ-ERBB2 is abnormally expressed in breast cancer cells. However, the role and mechanism of circ-ERBB2 in HER2-positive breast cancer are still unknown.

**Methods:**

The circ-ERBB2 expressions in the tumor tissues of HER2-positive breast cancer patients were tested using quantitative real-time PCR. The circ-ERBB2 function was investigated by cell counting kit 8 assay, Transwell, flow cytometry and Western blot. Mechanistically, fluorescence in situ hybridization, RNA immunoprecipitation, RNA pull-down and dual-luciferase reporter gene assays were conducted to confirm the interaction between circ-ERBB2 and miR-136-5p or miR-198 in HER2-positive breast cancer cells.

**Results:**

Circ-ERBB2 was elevated in the tumor tissues of HER2-positive breast cancer patients. Functionally, the interference with circ-ERBB2 repressed HER2-positive breast cancer cell proliferation, migration, invasion and accelerated cell apoptosis. Furthermore, the mechanistic analysis corroborated that circ-ERBB2 acted as a competing endogenous RNA for miR-136-5p or miR-198 to relieve the repressive influence of miR-136-5p or miR-198 on its target transcription factor activator protein 2C (TFAP2C). Meanwhile, in vivo assays further corroborated the oncogenic function of circ-ERBB2 in HER2-positive breast cancer.

**Conclusions:**

Circ-ERBB2 accelerated HER2-positive breast cancer progression through the circ-ERBB2/miR-136-5p/TFAP2C axis or the circ-ERBB2/miR-198/TFAP2C axis.

**Supplementary Information:**

The online version contains supplementary material available at 10.1186/s12967-021-03114-8.

## Introduction

Breast cancer is a pivotal cause of cancer-related deaths in women [1]. Human epidermal growth factor receptor 2 (HER2) is a proto-oncogene [2] and is overexpressed in nearly 20% of breast cancer patients, which leads to high recurrence rates and poor prognosis of HER2-positive breast cancer [3]. Thus, elucidating the molecular mechanism of the elevated HER2 in HER2-positive breast cancer is momentous to ameliorate HER2-positive breast cancer .

Circular RNAs (circRNAs) are tissue-specific and conserved endogenous non-coding RNAs that are enriched in mammalian cells [4, 5]. Due to the specificity of circRNAs under different pathological states, they are usually chosen as biomarkers for breast cancer diagnosis and treatment. For instance, Yang *et al*. corroborated that circAGFG1 is elevated and its expression is interrelated to the clinical stage and poor prognosis of triple-negative breast cancer patients [6]; Qu *et al*. demonstrated that silencing circRNA-CER in breast cancer cells restrains cell proliferation and migration, hinting that circRNA-CER might be an underlying biomarker for breast cancer prediction [7]. However, there are few studies on the circRNAs functions in HER2-positive breast cancer.

Circ-ERBB2, also named hsa_circ_0043459, has attracted our attention due to its function in tumor regulation. In gastric cancer, the elevated circ-ERBB2 enhances tumor growth and reduces overall survival in patients, and circ-ERBB2 exerts a carcinogenic effect in gastric cancer cells [8]; in lung cancer, propofol therapy represses the lung cancer cell proliferation and invasion via lessening circ-ERBB2, which provides a novel target for lung cancer treatment [9]. In the current research, we searched through the circBase database (http://www.circbase.org/) and discovered that circ-ERBB2 was expressed in human breast cancer cells MCF7. Crucially, a previous study also expounds that circ-ERBB2 is abnormally expressed in breast cancer cells [10]. What similar to this finding, our studies authenticated that circ-ERBB2 was highly expressed in HER2-positive breast cancer tissues and we further corroborated that the interference with circ-ERBB2 repressed HER2-positive breast cancer cell proliferation, migration, invasion and accelerated cell apoptosis.

Increasing evidence confirms that circRNAs act as miRNA sponges to participate in breast cancer development. Wu *et al*. corroborated that circIRAK3 sponges miR-3607 to accelerate breast cancer metastasis [11]; Liang *et al*. authenticated that circKDM4C is identified as a sponge for miR-548p and restrains breast cancer progression and attenuates doxorubicin resistance of breast cancer [12]. Importantly, we previously confirmed circ-ERBB2 was mainly located in the cytoplasm of HER2-positive breast cancer cells through fluorescence in situ hybridization, implying that circ-ERBB2 might function as the miRNAs sponge in HER2-positive breast cancer. Previous researches clarified that miR-136-5p, miR-564, miR-198, miR-892b, miR-890 and miR-377-3p are abnormally expressed in breast cancer [13–18]. Thus, these six miRNAs were chosen as the main miRNAs targeted by circ-ERBB2 in HER2-positive breast cancer in this research. Transcription factor activator protein 2C (TFAP2C), also known as AP-2γ, mediates the breast cancer occurrence and is a promising marker for predicting the prognosis of patients with HER2-positive breast cancer [19]. Meanwhile, the online bioinformatics software analysis displayed that TFAP2C might be the downstream target for miR-136-5p or miR-198 (among the above six miRNAs). Therefore, TFAP2C became one of the key molecules in this study.

In this research, we identified the abnormally elevated circ-ERBB2 in HER2-positive breast cancer and authenticated that the interference with circ-ERBB2 repressed tumor cell growth. Combined with the discovery that circ-ERBB2 was localized in the cytoplasm, we continued to investigate whether circ-ERBB2 exerted a regulatory function in HER2-positive breast cancer through the competing endogenous RNA (ceRNA) mechanism.

## Materials and methods

### Clinical samples

All clinical samples were gathered from the First Affiliated Hospital of Nanchang University. A total of 23 HER2-positive breast cancer tissues and 20 HER2-negative breast cancer tissues and the paired adjacent normal tissues were included in the current study. All clinical samples were taken from patients with diagnosed breast cancer who had not received any treatment prior to the sample collection. This study was approved by the Ethics Committee of the First Affiliated Hospital of Nanchang University and with the written consent of all participants. The correlation between circ-ERBB2 expression and clinical characteristics in breast cancer patients was presented in Table 1.

**Table 1:** Correlation between circ-ERBB2 expression and clinical characteristics in breast cancer patients

Clinicopathologic parameters	N	circ-ERBB2 expression		*p-*value
		Low (%)	High (%)	
Age (years)				
≤ 40	8	3 (37.5%)	5 (62.5%)	0.750
> 40	35	18 (51.4%)	17 (48.6%)	
Tumor size (cm)				
≤ 2	30	13 (43.3%)	17 (56.7%)	0.332
> 2	13	8 (61.5%)	5 (38.5%)	
Lymph node metastasis				
Negative	20	14 (70.0%)	6 (30.0%)	0.010*
Positive	23	7 (30.4%)	16 (69.6%)	
Histological grade				
I	11	7 (63.6%)	4 (36.4%)	0.214
II	14	8 (57.1%)	6 (42.9%)	
III	18	6 (33.3%)	12 (66.7%)	
TNM stage				
I	10	8 (80.0%)	2 (20.0%)	0.090
II	16	8 (50.0%)	8 (50.0%)	
III	13	4 (30.8%)	9 (69.2%)	
IV	4	1 (25.0%)	3 (75.0%)	
HER2 status				
Negative	20	15 (75.0%)	5 (25.0%)	0.001**
Positive	23	6 (26.1%)	17 (73.9%)	

TNM: tumor-node-metastasis

*P<0.05

**P<0.01

### Cell culture

Human breast epithelial cells MCF10A, HER2-positive breast cancer cells SKBR3 and BT-474 and the HER2-negative breast cancer cells MDA-MB-231 and MDA-MB-468 were from American Type Culture Collection (ATCC, Manassas, VA, USA).

MCF10A cells were put in Dulbecco’s Modified Eagle Medium: Nutrient Mixture F-12 (DMEM/F12) with 5% donor horse serum, EGF (20 ng/ml), insulin (10 μg/ml), hydrocortisone (0.5 μg/ml) and cholera toxin (100 ng/ml).

SKBR3 and BT-474 cells were put in DMEM with the addition of 10% FBS. The identification of HER2-positive breast cancer cells was conducted using Western blot and flow cytometry.

MDA-MB-231 and MDA-MB-468 cells were cultured in Dulbecco’s modified Eagle medium (DMEM) with 10% fetal bovine serum (FBS, Gibco, USA). All the cells were cultured at 37 °C, 5% CO_2_.

### Cell transfection

The small interfering RNA (si-RNA) targeting circ-ERBB2 (si-circ-ERBB2), miR-136-5p mimic, miR-136-5p inhibitor, miR-198 mimic and miR-198 inhibitor were synthesized by a biotechnology company (GenePharma, Shanghai, China).

For the SKBR3 and BT-474 cell transfection, the cells were seeded into 6-well plates and cultured overnight to about 75% confluence. Then si-circ-ERBB2, miR-136-5p mimic, miR-136-5p inhibitor, miR-198 mimic and miR-198 inhibitor were transfected into the cells at a dose of 40 nM using Lipofectamine 2000 (ThermoFisher Scientific, WA, USA). After 48 h, the transfection efficiency was checked using quantitative real-time PCR (qRT-PCR).

### qRT-PCR

In accordance with the scheme described in the previous literature [20], the qRT-PCR assay was conducted. Briefly, Trizol reagent (Invitrogen, California, USA) was carried out to isolate the total RNA from breast cancer tissues and cells. After the quality of RNA was verified by Nanodrop 2000 spectrophotometer (ThermoFisher Scientific), the RNA was reversely transcribed into cDNA using Primescript™ RT reagent (TaKaRa, Dalian, China), and the reverse transcription of miR-136-5p, miR-564, miR-198, miR-892b, miR-890 and miR-377-3p and U6 was conducted using Mir-X™ miRNA First-Strand Synthesis kit (Clontech, California, USA). Then real-time PCR was run on Light Cycler 480 II Real-Time PCR System (Roche, Switzerland) with SYBR Premix Ex Taq TM II Kit (TaKaRa). GAPDH and U6 were the endogenous controls. The 2^−ΔΔCt^ method was conducted to test the relative expression of different molecules.

### Cell counting kit 8 (CCK-8) assay

The SKBR3 and BT-474 cell proliferation was evaluated using CCK-8 assay. SKBR3 and BT-474 cells (1 × 10^4^) with different transfection were seeded in 96-well plates and cultured for 24, 48, 72 and 96 h. After that, CCK-8 solution (10 μl, Beyotime Biotechnology, Shanghai, China) was added and the incubation was continued for 2 h. The absorbance was tested at 450 nm under a microplate spectrophotometer (ThermoFisher Scientific).

### Transwell assay

For the cell migration, SKBR3 and BT-474 cells were put in the upper chamber of each insert (Corning, Lowell, MA, USA) with the noncoated membrane. The lower chambers were added 1% FBS. After 24 h of culture, the upper surface of the membrane was removed and the cells on the lower surface were stained using 0.1% crystal violet (Sigma, Missouri, USA) for 30 min.

For the cell invasion, we conducted the matrigel chambers (BD Biosciences, CA, USA) following the manufacturer's standard procedure. Firstly, we harvested the SKBR3 and BT-474 cells and then resuspended them in a serum-free medium. Subsequently, we transferred the cells to the hydrated matrigel chamber. The bottom chamber was incubated in DMEM containing 10% FBS overnight. Next, we scratched the cells on the upper surface and fixed the invasive cells on the lower surface and stained them with 0.1% crystal violet for 30 min.

### Flow cytometric analysis

Flow cytometry was carried out to quantify the SKBR3 and BT-474 cell apoptosis as the method described in the previous literature with minor changes [21], the cells were cultured in 6-well plates for 24 h. Then the cells were harvested and washed twice with PBS, and were incubated with fluorescein isothiocyanate (FITC)-Annexin V (5 μl) at room temperature (RT) for 10 min and were stained with PI solution (5 μl) in dark for 10 min. The SKBR3 and BT-474 cell apoptosis was estimated using a Gallios flow cytometer (Beckman, CA, USA).

For the identification of HER2-positive breast cancer cells, the HER2 expression was assessed using flow cytometry [22]. The cells were stained with antibodies against HER2 (HER2-FITC, CellSearch tumor phenotyping reagent HER2/neu) following the reagent manufacturer's standard procedures. The HER2 expression was determined by MACSQuant flow cytometer with the MACSQuant Analyzer 10 software (Miltenyi Biotec GmbH, Bergisch Gladbach).

### Western blot analysis

After the breast cancer tissues and cells were harvested, the RIPA lysis buffer (Beyotime Biotechnology) with PMSF was conducted to isolate the total protein. Then a BCA Protein Assay Kit (Beyotime Biotechnology) was carried out to quantify the protein concentrations. A total of 25 μg proteins were separated using sodium dodecyl sulfate–polyacrylamide gel electrophoresis and electro-transferred into PVDF membranes (Millipore, Massachusetts, USA). The above membranes were blocked using 5% skimmed milk and then incubated with the primary antibodies, such as anti-TFAP2C (also named AP2 gamma, 1/25000 dilution, Abcam), anti-HER2 (1/1000 dilution, Abcam), anti-Akt (1/500 dilution, Cell Signaling Technology), anti-p-Akt (Ser473, 1/500 dilution, Cell Signaling Technology), anti-ERK (1/1000 dilution, Abcam), anti-p-ERK (ERK1/2, Thr202/Tyr204, 1/1000 dilution, Santa Cruz Biotechnology) and anti-GAPDH (1/1000 dilution, Abcam) overnight at 4 °C. Then the membranes were incubated with a secondary antibody (1/2000 dilution, Abcam) at RT for 1 h. All the bands were visualized using enhanced chemiluminescence (ECL) reagent (ThermoFisher Scientific).

### Fluorescence in situ hybridization (FISH)

After harvesting SKBR3 and BT-474 cells, the cells were washed with PBS and fixed with 4% paraformaldehyde for 15 min and made into 4 μm slices. The slices were incubated with proteinase K for 15 min at RT and incubated with 0.07% streptavidin solution. Subsequently, a Fluorescent in Situ Hybridization Kit (RiboBio, Guangzhou, China) was conducted to analyze the cellular localization of circ-ERBB2 in accordance with the standard procedure for reagent manufacturers and DAPI (Beyotime Biotechnology) was added to stain the SKBR3 and BT-474 cell nucleus. All the images were acquired using a Zeiss LSM 700 confocal microscope (Carl Zeiss, Oberkochen, Germany).

### RNA immunoprecipitation (RIP)

RIP analysis was carried out using Magna RIP RNA-Binding Protein Immunoprecipitation Kit (Millipore). SKBR3 and BT-474 cells were re-suspended with RIP lysis buffer with protease and RNase inhibitors. Then cell lysates were incubated with anti-IgG or anti-Ago2 (Abcam, USA)-coated beads with rotation at 4 °C overnight. The RNA that was bound to the magnetic beads was extracted, the circ-ERBB2 expression was quantified using qRT-PCR.

### RNA pull-down

Biotin-coupled circ-ERBB2 and a negative control (NC) probe were synthesized from the biological company (RiboBio). SKBR3 and BT-474 cells were harvested and lysed. Circ-ERBB2 probe was further incubated with streptavidin-coated magnetic beads (ThermoFisher Scientific) at RT for approximately 2 h to produce probe-coated beads. Subsequently, the above cell lysate was incubated with the probe-coated beads at 4 °C overnight. The beads were then washed and the RNA complexes that bound to the beads were eluted by wash buffer. The expressions of the bound miRNAs (miR-136-5p, miR-564, miR-198, miR-892b, miR-890 and miR-377-3p) in the pull-down complexes were tested by qRT-PCR.

### Dual-luciferase reporter gene assay

The predicted binding sites between circ-ERBB2 and miR-136-5p, circ-ERBB2 and miR-198, miR-136-5p and TFAP2C, miR-198 and TFAP2C were cloned into a dual-luciferase reporter vector (ThermoFisher Scientific). The above dual-luciferase reporter vector and miR-136-5p mimic or miR-198 mimic were co-transfected into SKBR3 and BT-474 cells. Forty-eight hours later, the luciferase activity was quantified using the Dual-Luciferase Reporter Assay Kit (ThermoFisher Scientific).

### Xenograft mouse model

Sixteen female Balb/cnu/nu immune-deficient mice (4 weeks old) were purchased from Charles River (Burlington, MA, USA). Mice were grouped: Lenti-sh-NC and Lenti-sh-circ-ERBB2, and eight mice were randomly assigned to each group. For the mice in the Lenti-sh-circ-ERBB2 group, after BT-474 cell lines with lenti-sh-circ-ERBB2 stable transformation were constructed, the BT-474 cells (2 × 10^5^ cells in 30 μl PBS) were mixed with 25 μl Matrigel on ice. Then the cell suspension was quickly injected into the fourth mammary fat pad of mice. The mouse tumor volume was monitored using caliper measurements of tumor length (L) and width (W) according to the formula LW^2^/2 weekly and lasted for 4 weeks and the tumor weight was monitored. Four weeks later, mice were sacrificed and the tumor tissues, lung tissues and heart tissues were collected for the follow-up research. All the animal experiments were carried out in accordance with the Animal Care and Use Committee of the First Affiliated Hospital of Nanchang University.

### Hematoxylin–eosin (HE) staining

After obtaining the heart and lung tissues of the mice, 10% paraformaldehyde was applied to fix the tissues and then made into 4 μm slices. Then, the slices were stained with hematoxylin (Beyotime Biotechnology) for 5 min, followed by soaking in warm water (about 50 °C) for 5 min. The above slices were then stained with eosin solution (Beyotime Biotechnology) for 2 min. A microscope (Shanghai Optical Instrument Factory, Shanghai, China) was applied for photography and observation.

### Statistical analysis

All data were assessed by GraphPad Prism 5.0 and were expressed as mean ± standard deviation. Differences between groups were evaluated using Student’s *t*-test or one-way ANOVA test unless otherwise specified. Differences between groups were considered significant when the *P* < 0.05.

## Results

### Circ-ERBB2 is highly expressed in tumor tissues of HER2-positive breast cancer patients and positively correlated with TFAP2C expression

**Fig. 1:**
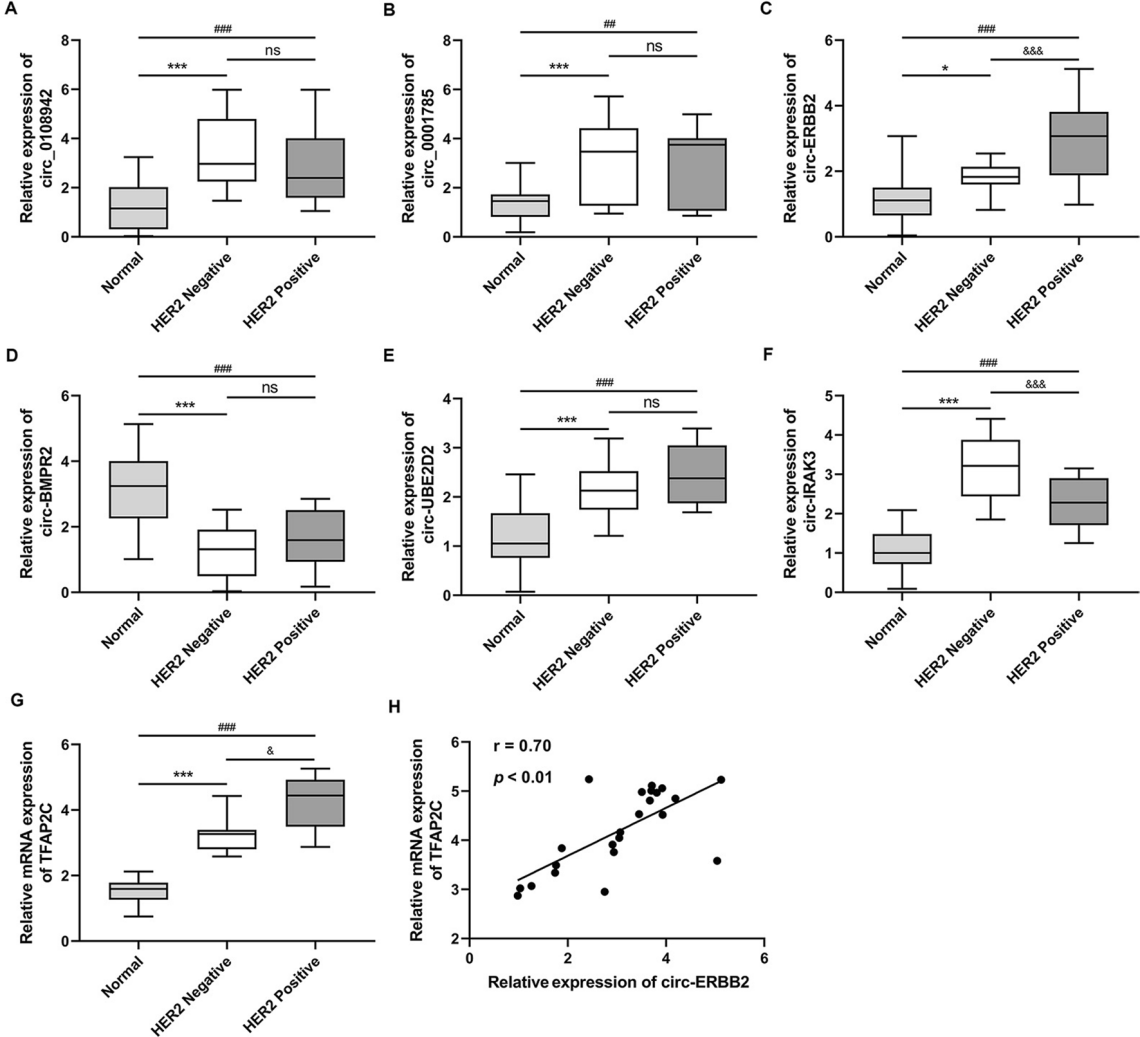
Analysis of circ-ERBB2 expression in the tumor tissues of HER2-positive breast cancer patients and its correlation with TFAP2C expression. HER2-positive (23 pairs) and HER2-negative (20 pairs) breast cancer tissues and adjacent normal tissues were collected. **A**–**F** Quantitative real-time PCR (qRT-PCR) was performed to measure the expressions of circ_0108942, circ_0001785, circ-ERBB2, circ-BMPR2, circ-UBE2D2 and circ-IRAK3 (the abnormal expressions of them that have been reported in breast cancer tissues). **A**, **B** Kruskal-Walllis test with Dunn’s post hoc; **C** Welched one-way ANOVA with Games-Howell’s post hoc; **D**–**F** one-way ANOVA with Sidak’s post hoc. **G** Analysis of the TFAP2C expression by qRT-PCR, Kruskal–Wallis test with Dunn’s post hoc. **H** Pearson correlation analysis of the correlation between circ-ERBB2 expression and TFAP2C expression in HER2-positive breast cancer tissues (r = 0.70, *P* < 0.01, n = 23), Pearson r correlation test. Data are represented as mean ± SD. *P < 0.05, ***P < 0.001 vs. Normal. ^&&&^*P* < 0.001 vs. HER2 Negative. ns: not significant

To quantify the abnormal expressions of the six circRNAs (circ_0108942, circ_0001785, circ-ERBB2, circ-BMPR2, circ-UBE2D2, circ-IRAK3) in breast cancer tissues that are reported in previous literature, the qRT-PCR results authenticated that in comparison with the HER2 Negative group, circ-ERBB2 expression was elevated in the HER2 Positive group and circ-IRAK3 was decreased, and the other four circRNAs had no remarkable changes (Fig. 1A–F). Thus, circ-ERBB2 and circ-IRAK3 were chosen for our subsequent researches. Furthermore, the TFAP2C was increased in the HER2 Positive group compared with the HER2 Negative group. Meanwhile, in patients with HER2-positive breast cancer, we discovered a positive correlation between circ-ERBB2 and TFAP2C expressions (Fig. 1H). Thus, circ-ERBB2 was selected as the main circRNA in this research. Conclusively, our data expounded that circ-ERBB2 was increased in tumor tissues of HER2-positive breast cancer patients and its expression was positively correlated with TFAP2C expression.

### Interfering circ-ERBB2 restrains SKBR3 and BT-474 cell proliferation, migration, invasion and accelerates cell apoptosis

**Fig. 2:**
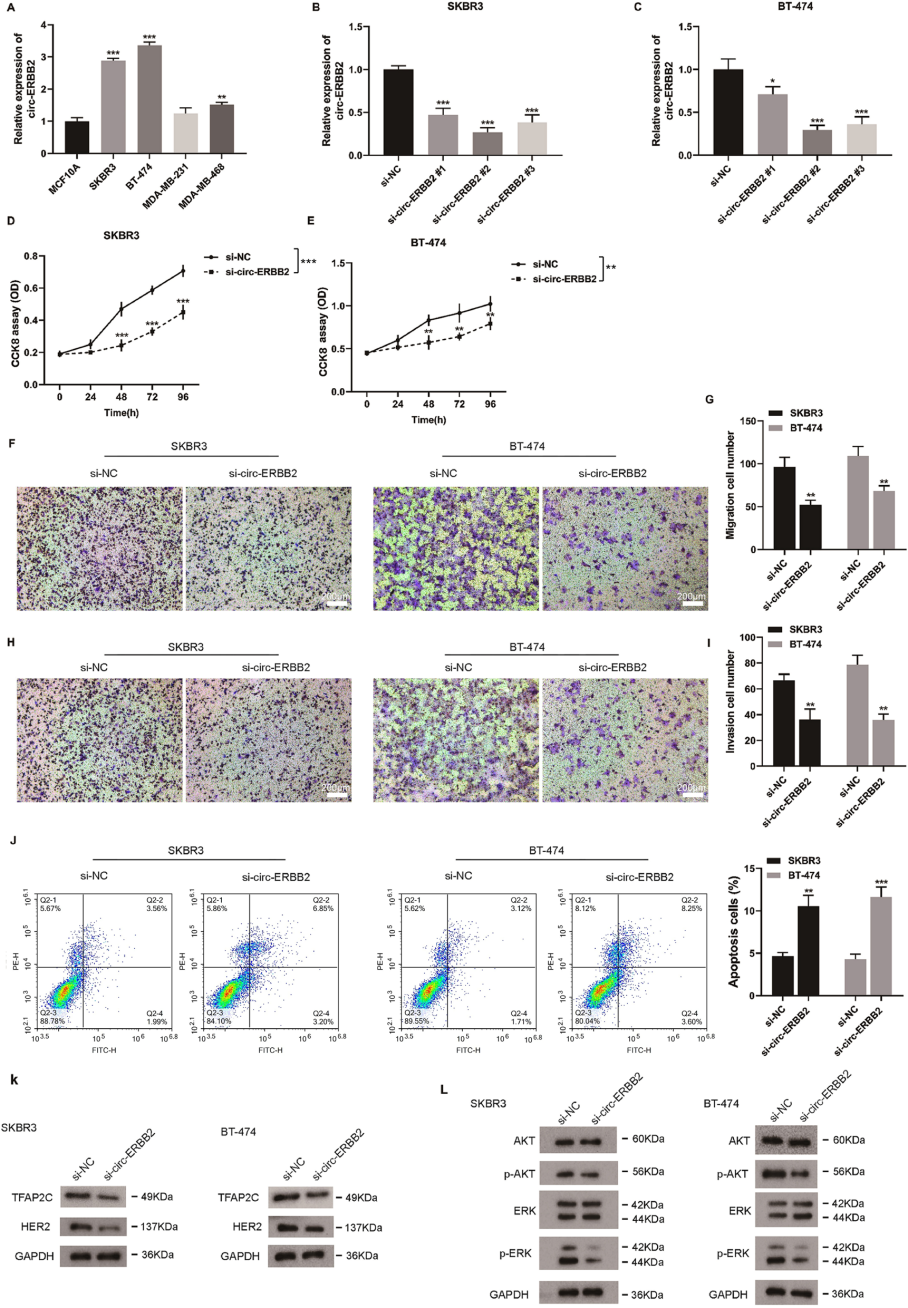
Effect of circ-ERBB2 on the proliferation, migration, invasion and apoptosis of SKBR3 and BT-474 cells. **A** Detection of circ-ERBB2 levels in human breast epithelial cells MCF10A, HER2-positive breast cancer cells SKBR3 and BT-474 and HER2-negative breast cancer cells MDA-MB-231 and MDA-MB-468, one-way ANOVA with Tukey’s post hoc. **B**, **C** si-circ-ERBB2 #1, si-circ-ERBB2 #2 and si-circ-ERBB2 #3 were separately transfected into SKBR3 and BT-474 cells, one-way ANOVA with Tukey’s post hoc. The transfection efficiency of si-circ-ERBB2 was assessed by qRT-PCR. **D**, **E** si-circ-ERBB2 #2 was transfected into SKBR3 and BT-474 cells. Cell counting kit 8 (CCK-8) assay was applied to analyze the proliferation of SKBR3 and BT-474 cells, two-way ANOVA for group comparison, multiple t-test to compare the same time between two groups. **F**–**I** The cell migration and invasion were measured by Transwell assay (scale bar = 200 μm), **G**, **I** or **J** unpaired two-tailed t-test. **J** Flow cytometry was conducted to detect the apoptosis of SKBR3 and BT-474 cells, values are the mean ± SD of three independent experiments. **K**, **L** The protein levels of TFAP2C, HER2 and the downstream signaling pathway proteins Akt, p-Akt, ERK and p-ERK were measured by Western blot analysis. Data are represented as mean ± SD. *P < 0.05, **P < 0. 01, ***P < 0.001 vs. si-NC. ***P < 0.001 vs. MCF10A. NC: negative control

To investigate the circ-ERBB2 biological function on HER2-positive breast cancer, we firstly assessed the circ-ERBB2 expressions in HER2-positive breast cancer and HER2-negative breast cancer cell lines. As expected, compared with the human breast epithelial cells MCF10A, circ-ERBB2 expression was enhanced in the HER2-positive breast cancer cells SKBR3 and BT-474 and was lessened in the HER2-negative breast cancer cells MDA-MB-468 and the HER2-positive breast cancer cells SKBR3 and BT-474 were applied for the follow-up study (Fig. 2A). After si-circ-ERBB2 #1, si-circ-ERBB2 #2 and si-circ-ERBB2 #3 were separately transfected into SKBR3 and BT-474 cells, the transfection efficiency was presented in Fig. 2B, C. The si-circ-ERBB2 #2 had the best interference effect, so it was selected for our research. Subsequently, CCK-8 analysis expounded that the decreased circ-ERBB2 repressed the SKBR3 and BT-474 cell proliferation (Fig. 2D, E). Similarly, the Transwell results corroborated that the interference with circ-ERBB2 suppressed the SKBR3 and BT-474 cell migration and invasion (Fig. 2F–I). Meanwhile, the loss of circ-ERBB2 accelerated the SKBR3 and BT-474 cell apoptosis (Fig. 2J). Based on these findings, the protein levels of TFAP2C, HER2 and the downstream signaling pathway proteins Akt, p-Akt, ERK and p-ERK were further assessed. As displayed in Fig. 2K, L, the interference with circ-ERBB2 lessened the TFAP2C, HER2, p-Akt and p-ERK protein levels. The expression of HER2 in HER2-positive breast cancer cells was determined using Western blot (Fig. 2K). Moreover, the HER2 expression was further verified in HER2-positive breast cancer cells by flow cytometry (Additional file 1: Figure S1A). In general, the interference with circ-ERBB2 repressed HER2-positive breast cancer cell proliferation, migration, invasion and accelerated cell apoptosis.

### Verification of interactions between circ-ERBB2 and miR-136-5p, circ-ERBB2 and miR-198 in SKBR3 and BT-474 cells

**Fig. 3:**
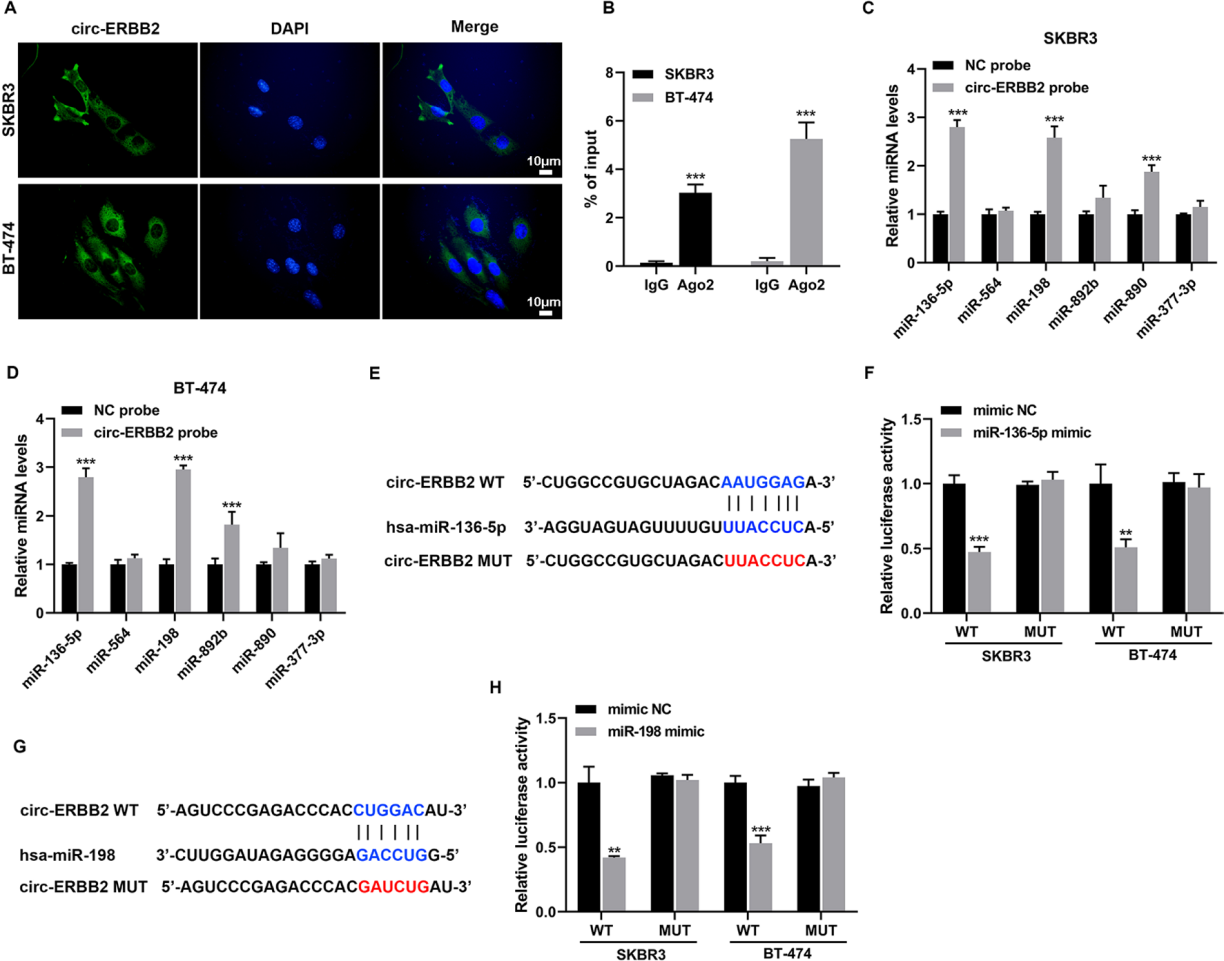
Interaction between circ-ERBB2 and miR-136-5p, circ-ERBB2 and miR-198 in SKBR3 and BT-474 cells. **A** Fluorescence in situ hybridization (FISH) was applied to analyze the localization of circ-ERBB2 in SKBR3 and BT-474 cells (scale bar = 10 μm). **B** The binding of circ-ERBB2 to Ago2 was verified by RNA immunoprecipitation (RIP), unpaired two-tailed t-test. **C**, **D** RNA pull-down assay was conducted to investigate the interaction between circ-ERBB2 and miR-136-5p, miR-564, miR-198, miR-892b, miR-890 and miR-377-3p (all of them are reported in breast cancer and might be regulated by circ-ERBB2), unpaired two-tailed t-test. **E** Online bioinformatics software (Circular RNA interactome) analysis revealed that there were binding sites between circ-ERBB2 and miR-136-5p. **F** The interaction between circ-ERBB2 and miR-136-5p was confirmed by dual-luciferase reporter gene assay, unpaired two-tailed t-test. **G** Online bioinformatics software (Circular RNA interactome) analysis predicted that there were binding sites between circ-ERBB2 and miR-198. **H** Dual-luciferase reporter gene assay was applied to verify the interaction between circ-ERBB2 and miR-198, unpaired two-tailed t-test. **P < 0.01 vs. mimic NC. Data are represented as mean ± SD. ***P < 0.001 vs. IgG, NC probe or mimic NC. WT: wild type; MUT: mutant type

Based on our confirmation of the circ-ERBB2 critical function in SKBR3 and BT-474 cells, we continue to investigate whether circ-ERBB2 functioned in cells by miRNA adsorption through a ceRNA mechanism. Firstly, the circ-ERBB2 localization in SKBR3 and BT-474 cells was assessed and the results demonstrated that circ-ERBB2 was mainly located in the cytoplasm of SKBR3 and BT-474 cells, hinting that circ-ERBB2 might function as a "sponge" for miRNAs in the cytoplasm (Fig. 3A). The binding of circ-ERBB2 to Ago2 was verified by RIP and the results clarified that Ago2 antibody enriched circ-ERBB2, implying that circ-ERBB2 was incorporated into the RNA-induced silencing complex (Fig. 3B). Meanwhile, we used the specific probe of circ-ERBB2 for RNA pull-down assay to verify the miRNAs (miR-136-5p, miR-564, miR-198, miR-892b, miR-890 and miR-377-3p) that have been reported in breast cancer and might be regulated by circ-ERBB2. As presented in Fig. 3C, D, in SKBR3 and BT-474 cells, both miR-136-5p and miR-198 were enriched in the pull-down complex of circ-ERBB2. Hence, miR-136-5p and miR-198 were chosen as the main miRNAs. As expected, both miR-136-5p and miR-198 negatively regulated the luciferase activity of circ-ERBB2 in SKBR3 and BT-474 cells (Fig. 3E–H). Meanwhile, miR-136-5p and miR-198 were lowly expressed in the HER2-positive breast cancer cells (Additional file 1: Figure S1B). In summary, circ-ERBB2 interacted with miR-136-5p or miR-198 in SKBR3 and BT-474 cells.

### Verification of targeted regulation of TFAP2C by miR-136-5p and miR-198 in SKBR3 and BT-474 cells

**Fig. 4:**
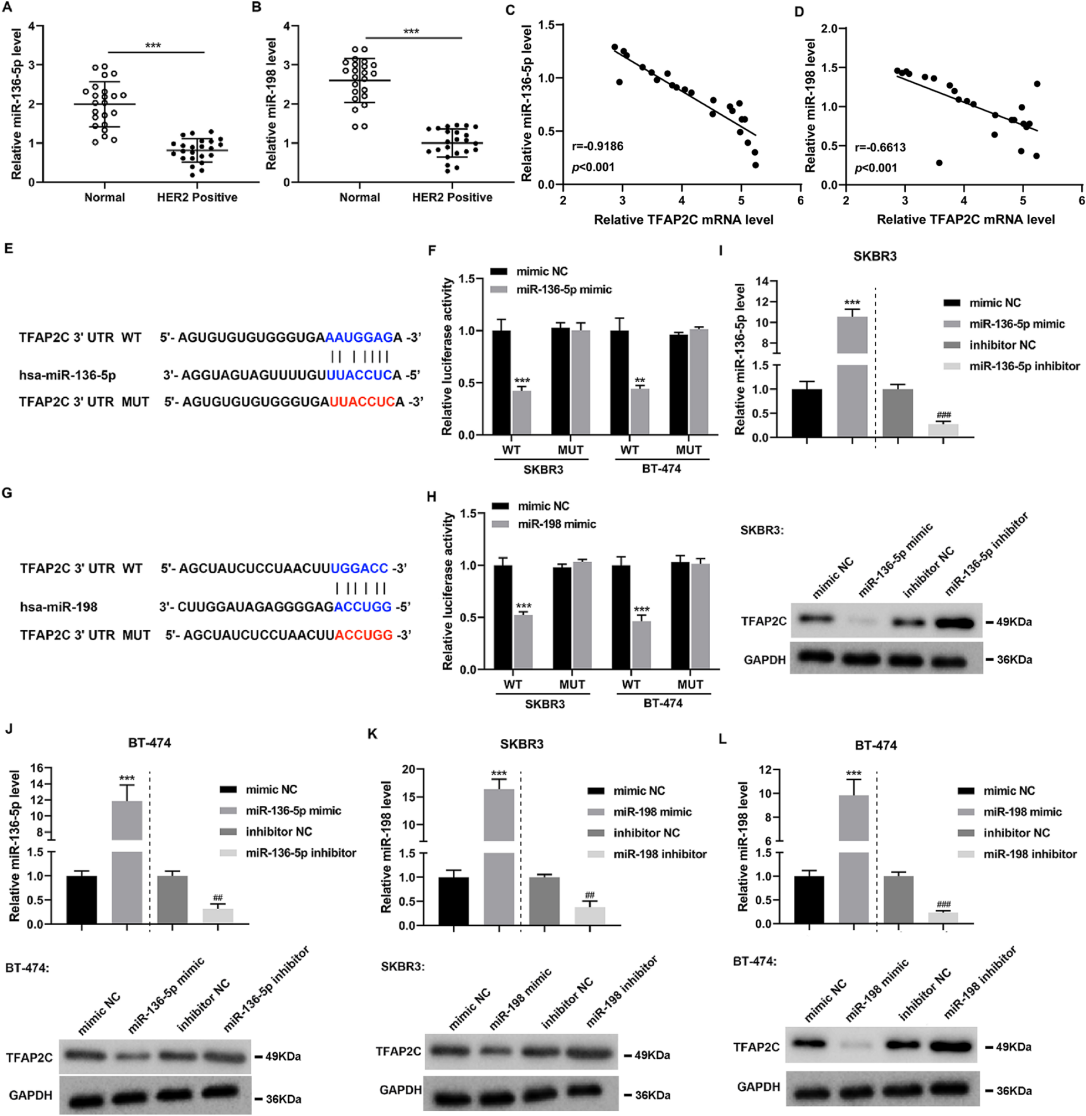
The targeted regulation of TFAP2C by miR-136-5p and miR-198 in SKBR3 and BT-474 cells. **A**, **B** Detection of miR-136-5p and miR-198 expressions in HER2-positive breast cancer tissues and the adjacent normal tissues by qRT-PCR, unpaired two-tailed t-test. **C**, **D** Pearson correlation analysis of the correlation between miR-136-5p expression and TFAP2C expression (r =-0.9186, *P* < 0.001, n = 23), miR-198 expression and TFAP2C expression (r =-0.6613 , *P* < 0.001, n = 23) in HER2-positive breast cancer tissues, Pearson r correlation test. **E** The binding sites between miR-136-5p and TFAP2C were predicted by online bioinformatics software (RNAInter). **F** Dual-luciferase reporter gene assay was applied to prove the interaction between miR-136-5p and TFAP2C, unpaired two-tailed t-test. **G** Online bioinformatics software (RNAInter) analysis discovered that there were binding sites between miR-198 and TFAP2C. **H** The interaction between miR-198 and TFAP2C was confirmed by a dual-luciferase reporter gene assay, unpaired two-tailed t-test. **I**–**L** miR-136-5p mimic, miR-136-5p inhibitor, miR-198 mimic or miR-198 inhibitor was transfected into SKBR3 and BT-474 cells. The transfection efficiency was verified by qRT-PCR and the protein level of TFAP2C was quantified by Western blot, unpaired two-tailed t-test. Data are represented as mean ± SD. ***P < 0.001 vs. Normal or mimic NC. ^##^*P* < 0.01, ^###^*P* < 0.001 vs. inhibitor NC

Next, the miR-136-5p and miR-198 expressions were examined in HER2-positive breast cancer tissues and the adjacent normal tissues. As displayed in Fig. 4A, B, in comparison with the Normal group, miR-136-5p and miR-198 were lowly expressed in HER2-positive breast cancer tissues. In patients with HER2-positive breast cancer, the miR-136-5p or miR-198 was both negatively correlated with the TFAP2C expression (Fig. 4C, D). Furthermore, both miR-136-5p and miR-198 negatively regulated the luciferase activity of TFAP2C (Fig. 4E–H). After the transfection of miR-136-5p mimic, miR-136-5p inhibitor, miR-198 mimic or miR-198 inhibitor into SKBR3 and BT-474 cells, the transfection efficiency data were presented in Fig. 4I–L. Furthermore, both miR-136-5p and miR-198 negatively regulated the TFAP2C protein level (Fig. 4I–L). In general, both miR-136-5p and miR-198 targeted TFAP2C and negatively regulated its protein level.

### Circ-ERBB2 regulates TFAP2C through miR-136-5p or miR-198 to affect the function of SKBR3 and BT-474 cells

**Fig. 5:**
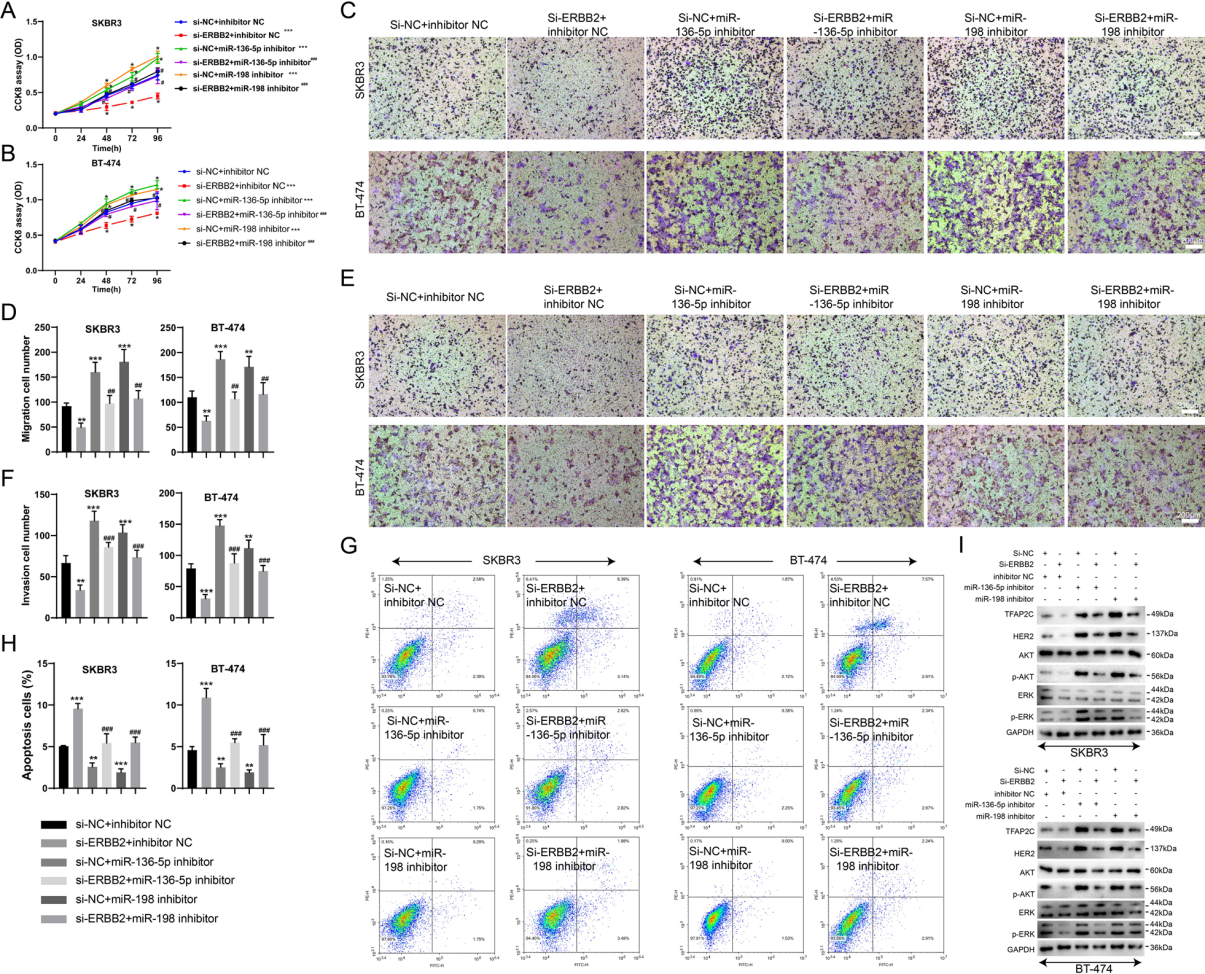
Circ-ERBB2 influenced the proliferation, invasion, migration and apoptosis of SKBR3 and BT-474 cells through the circ-ERBB2/miR-136-5p/TFAP2C axis or the circ-ERBB2/miR-198/TFAP2C axis. si-circ-ERBB2 and/or miR-136-5p inhibitor, si-circ-ERBB2 and/or miR-198 inhibitor were separately transfected into SKBR3 and BT-474 cells. **A**, **B** The proliferation of SKBR3 and BT-474 cells was assessed by CCK-8 assay, two-way ANOVA combined with Tukey post-hoc. **C**–**F** The migration and invasion of SKBR3 and BT-474 cells were analyzed by Transwell assay (scale bar = 200 μm), **D** One-way ANOVA combined with LSD post-hoc, **F** One-way ANOVA combined with LSD post-hoc. **G**, **H** The apoptosis of SKBR3 and BT-474 cells was assessed by flow cytometry analysis, **H** One-way ANOVA combined with LSD post-hoc. **I** The protein levels of TFAP2C, HER2, Akt, p-Akt, ERK and p-ERK were detected by Western blot. Data are represented as mean ± SD. *P < 0.05, **P < 0.01 or ***P < 0.001 vs. si-NC + inhibitor NC. ^#^*P* < 0.05, ^##^*P* < 0.01 or ^###^*P* < 0.001 vs. si-ERBB2 + inhibitor NC

To illustrate whether circ-ERBB2 exerted its biological function through the circ-ERBB2/miR-136-5p/TFAP2C axis or the circ-ERBB2/miR-198/TFAP2C axis, we designed a series of rescue assays using miR-136-5p inhibitor or miR-198 inhibitor in SKBR3 and BT-474 cells. As displayed in Fig. 5A, B, the interference with circ-ERBB2 restrained the SKBR3 and BT-474 cell proliferation, while this restraint was reversed by the miR-136-5p knockdown or miR-198 knockdown. Similarly, the circ-ERBB2 knockdown weakened the invasion and migration abilities of SKBR3 and BT-474 cells, and the miR-136-5p knockdown or miR-198 knockdown reversed this trend (Fig. 5C–F). In contrast, the interference with circ-ERBB2 accelerated the SKBR3 and BT-474 cell apoptosis, while this trend was reversed by the miR-136-5p knockdown or miR-198 knockdown (Fig. 5G, H). Meanwhile, the circ-ERBB2 knockdown lessened the TFAP2C, HER2, p-Akt and p-ERK protein levels, and the miR-136-5p knockdown or miR-198 knockdown counteracted the impacts of circ-ERBB2 down-regulation on these protein levels (Fig. 5I). Collectively, circ-ERBB2 might function as a ceRNA for miR-136-5p or miR-198 to positively regulate the TFAP2C protein level, thus influencing the SKBR3 and BT-474 cell function.

### Verification of the circ-ERBB2 impact on tumor growth in vivo

**Fig. 6:**
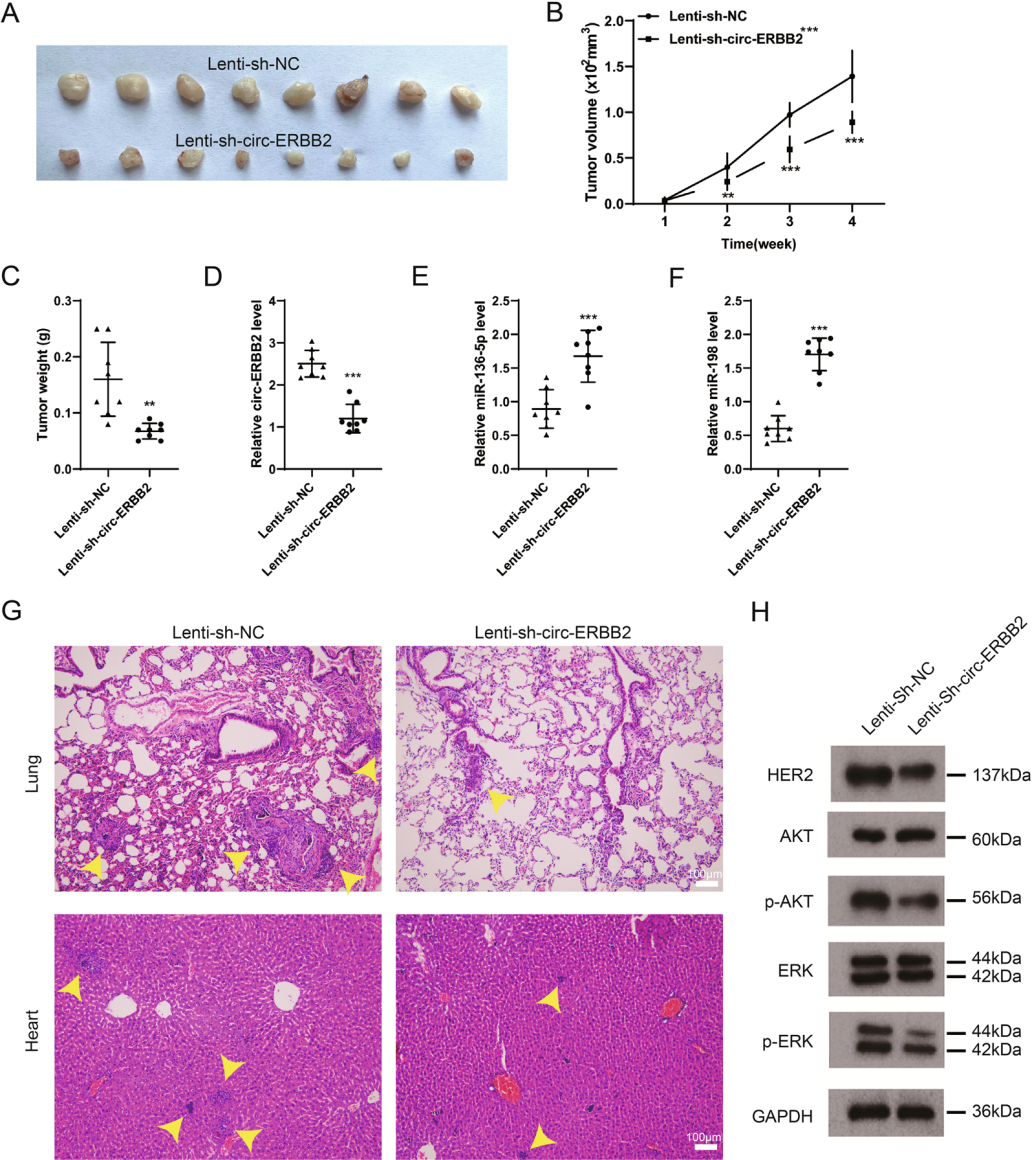
Influence of circ-ERBB2 on tumor growth in vivo. BT-474 cell lines with Lenti-sh-circ-ERBB2 stable transformation were constructed, and stable BT-474 cells (2 × 10^5^ cells in 30 μl PBS) were mixed with 25 μl Matrigel on ice, and then the cell suspension was quickly injected into the fourth mammary fat pad of 4-week-old female mice. **A**–**C** Detection of the tumor volume and tumor weight, **B** two-way ANOVA analysis; **C** unpaired two-tailed t-test. **D** qRT-PCR was applied to measure the expression of circ-ERBB2, unpaired two-tailed t-test. **E**, **F** Analysis of the miR-136-5p and miR-198 expressions by qRT-PCR, unpaired two-tailed t-test. **G** Hematoxylin–eosin (HE) staining was conducted to display the metastatic nodules of the lung and heart of mice (Scale bar = 100 μm). **H** The protein levels of HER2, Akt, p-Akt, ERK and p-ERK were analyzed by Western blot. Data are represented as mean ± SD. **P < 0.01, ***P < 0.001 vs. Lenti-sh-NC

To estimate the oncogene function of circ-ERBB2 in vivo, the BT-474 stable cell line was mixed with matrigel on ice, and the cell suspension was quickly injected into the fourth mammary fat pad of female mice. In comparison with the Lenti-sh-NC group, the circ-ERBB2 knockdown reduced the tumor volume and weight (Fig. 6A–C). The interference efficiency of circ-ERBB2 was verified using qRT-PCR (Fig. 6D). Moreover, the circ-ERBB2 knockdown elevated both miR-136-5p and miR-198 expressions (Fig. 6E, F). Furthermore, the circ-ERBB2 knockdown led to a decrease of metastasis nodes in the lung and heart of mice (Fig. 6H). Meanwhile, in comparison with the Lenti-sh-NC group, the circ-ERBB2 knockdown lessened the HER2, p-Akt and p-ERK protein levels. To sum up, the circ-ERBB2 knockdown repressed the tumor growth in vivo.

## Discussion

Recently, circRNAs have become the research focus for their pivotal functions in various cancers. So far, some circRNAs have been identified as the biomarkers for breast cancer [23–25]. However, there are few reports about circRNAs in HER2-positive breast cancer. Here, we assessed the expressions of six circRNAs that have been reported to be abnormally expressed in breast cancer: circ_0108942, circ_0001785, circ-ERBB2, circ-BMPR2, circ-UBE2D2, circ-IRAK3 [10, 11, 26–28]. Through the preliminary screening of qRT-PCR, we focused on a novel circRNA: circ-ERBB2, which was abnormally high expressed in tumor tissues of patients with HER2-positive breast cancer and was interrelated to Lymph node metastasis and HER2 status in patients. Functionally, circ-ERBB2 knockdown restrained HER2-positive breast cancer cell proliferation, migration, invasion and accelerated cell apoptosis. Mechanically, circ-ERBB2 positively regulated TFAP2C expression through sponging miR-136-5p or miR-198, thereby regulating HER2-positive breast cancer cell function. Generally, our data corroborated that circ-ERBB2 might act as an oncogene in HER2-positive breast cancer development and provide two novel regulatory axes for HER2-positive breast cancer: circ-ERBB2/miR-136-5p/TFAP2C and circ-ERBB2/miR-198/TFAP2C.

The ceRNA hypothesis suggests that non-coding RNA transcripts regulate target gene expression by competing for the same miRNA response element (MRE) [29, 30]. Accumulated evidence demonstrates that circRNAs act as miRNA sponges to regulate miRNA target gene expression in breast cancer. For instance, circ_100876 accelerates breast cancer cell proliferation and metastasis through sponging miR-361-3p [31]; circKIF4A functions as a ceRNA to accelerate the KIF4A expression by sponging miR-375, thus aggravating breast cancer [32]. In the present study, the FISH assay corroborated that circ-ERBB2 was mainly located in the cytoplasm of HER2-positive breast cancer cells, prompting that circ-ERBB2 might function as a miRNAs "sponge" in the cytoplasm. Critically, previous researches authenticate that miR-136-5p, miR-564, miR-198, miR-892b, miR-890 and miR-377-3p are abnormally expressed in breast cancer [13–18]. To further investigate whether circ-ERBB2 in HER2-positive breast cancer affected the cell growth and metastasis through one or more of the target miRNAs, we screened and validated the above six miRNAs. As expected, RIP, RNA pull-down and dual-luciferase reporter assays confirmed the binding of circ-ERBB2 to miR-136-5p or miR-198 in HER2-positive breast cancer cells. Thus, we supposed that circ-ERBB2 might exert its oncogenic function in HER2-positive breast cancer by sponging miR-136-5p or miR-198.

TFAP2C is a member of the AP2 transcription factor family and has pivotal regulation in cell proliferation, cell cycle and apoptosis [33]. TFAP2C is initially confirmed to function in embryonic development [34]. With the continuous research on the TFAP2C function, increasing evidence expounds that TFAP2C mediates breast cancer progression. For instance, Turner *et al*. confirmed that TFAP2C is highly expressed in human breast cancer and TFAP2C controls the breast cancer cell growth and differentiation [35]. Critically, Wu *et al*. demonstrated that TFAP2C drives the HER2-positive breast cancer cell proliferation and invasion and predicts the prognosis in patients with HER2-positive breast cancer [19]. In the present study, online bioinformatics software analysis displayed that TFAP2C might be the downstream target for miR-136-5p or miR-198 and this hypothesis was further confirmed using a dual-luciferase reporter gene assay. Meanwhile, our in-depth study clarified that both miR-136-5p and miR-198 negatively regulated the TFAP2C expression in HER2-Positive Breast Cancer cell lines, which was similar to the previous conclusions [36, 37]. Based on the above findings, we further demonstrated that the interference with circ-ERBB2 repressed HER2-positive breast cancer cell proliferation, invasion, migration and accelerated cell apoptosis via the miR-136-5p/TFAP2C axis or the miR-198/TFAP2C axis through the rescue assays. Accumulated evidence expounds that the Akt and ERK signaling pathways are interrelated to breast cancer progression and are regulated by circRNAs [38, 39]. Here, our study preliminarily corroborated that the circ-ERBB2 knockdown inactivated the Akt and ERK signaling pathways via the miR-136-5p/TFAP2C axis or the miR-198/TFAP2C axis. Moreover, in vivo assays further expounded that the circ-ERBB2 knockdown restrained the HER2-positive breast cancer growth and metastasis.

In conclusion, our results demonstrated that circ-ERBB2 was highly expressed in HER2-positive breast cancer and was interrelated to Lymph node metastasis and HER2 status in patients with HER2-positive breast cancer and the circ-ERBB2 knockdown repressed HER2-positive breast cancer cell growth, preliminarily confirming that circ-ERBB2 might be a novel oncogene in HER2-positive breast cancer and we further provided novel ceRNA regulatory pathways for HER2-positive breast cancer: circ-ERBB2/miR-136-5p/TFAP2C or circ-ERBB2/miR-198/TFAP2C. Our research might bring promising and valuable biomarkers for HER2-positive breast cancer treatment.

## Supplementary Information

**Additional file 1: Figure S1.** Analysis of the miR-136-5p and miR-198 expressions in the HER2-positive breast cancer cells and the verification of HER2-positive breast cancer cells. (A) Verification of HER2-positive breast cancer cells by flow cytometry. (B) Detection of the miR-136-5p and miR-198 expressions in the HER2-positive breast cancer cells using qRT-PCR. *P < 0.05, **P < 0.01, ***P < 0.001, ****P < 0.0001 vs. MCF10A.

## Data Availability

All data generated or analyzed during this study are included in this published article.
