# Supplementary figures and images for: Circular RNA circ-ERBB2 promotes HER2-positive breast cancer progression and metastasis via sponging miR-136-5p and miR-198
Source: J Transl Med. 2021 Nov 3;19:455. doi: 10.1186/s12967-021-03114-8 (PMC8564996; doi:10.1186/s12967-021-03114-8)

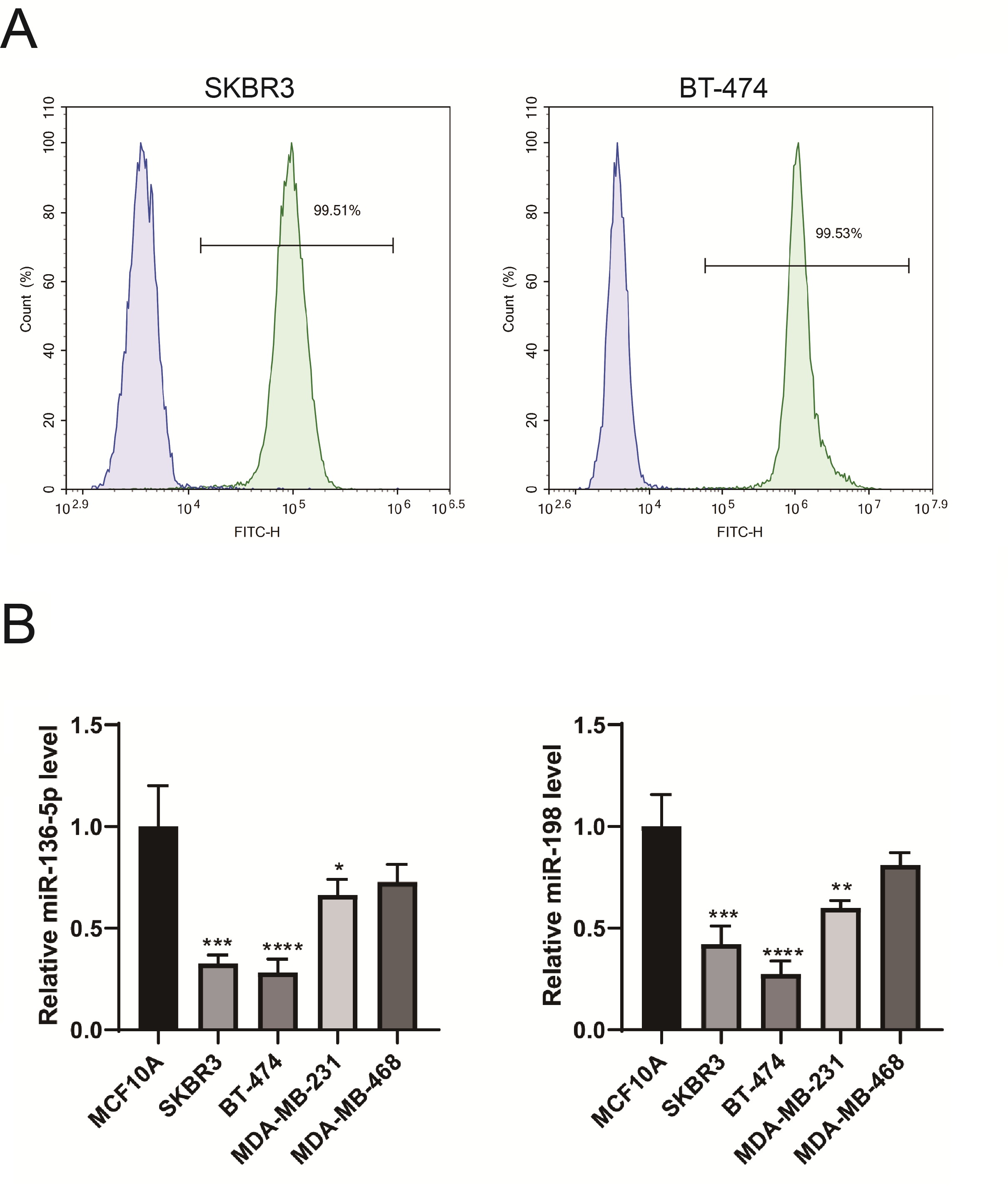

Supplement: Supplementary file 1 — Additional file 1: Figure S1. Analysis of the miR-136-5p and miR-198 expressions in the HER2-positive breast cancer cells and the verification of HER2-positive breast cancer cells. (A) Verification of HER2-positive breast cancer cells by flow cytometry. (B) Detection of the miR-136-5p and miR-198 expressions in the HER2-positive breast cancer cells using qRT-PCR. *P < 0.05, **P < 0.01, ***P < 0.001, ****P < 0.0001 vs. MCF10A. [file 12967_2021_3114_MOESM1_ESM.jpg]
